# A Novel Preparation Method of Electrically Conductive Adhesives by Powder Spraying Process

**DOI:** 10.3390/ma12172793

**Published:** 2019-08-30

**Authors:** Hongyu Dong, Xin Li, Yi Dong, Shaoqing Guo, Liangfu Zhao

**Affiliations:** 1 Institute of Coal Chemistry, Chinese Academy of Sciences, Taiyuan 030001, Shanxi, China; 2 College of Environment and Safety, Taiyuan University of Science and Technology, Taiyuan 030024, Shanxi, China

**Keywords:** electrically conductive adhesives, silver flakes, electrical resistivity

## Abstract

In a conventional electrically conductive adhesive (ECA) preparation process, typical ECAs are made by adding an appropriate amount of electrically conductive fillers, such as silver, into a polymer matrix, such as epoxy resin, to form a uniformly dispersed mixture by mixing and stirring operations. However, during the preparation process, secondary pollution and mass loss are caused by the vigorous mixture process. At the same time, the stirring operation introduces many small and stable bubbles, which affect the electrical conductivity of the ECAs. In light of these problems with the conventional preparation of ECAs, we developed a novel ECA preparation method that employs a powder spraying process: silver flakes are sprayed into the epoxy resin with a certain mass fraction to form formulated pastes. The as-prepared ECAs exhibited excellent properties compared with those prepared by the conventional process. This proves that the powder spraying process is feasible and superior to the conventional process.

## 1. Introduction

The interconnected technology of electronics and photovoltaic manufacturing has been dominated by tin–lead alloy solders since the very beginning [1,2,3]. Tin–lead alloy solders, which provide a conductive path to complete the circuit, have desirable electrical, mechanical, and thermal properties [4,5,6,7,8,9,10] during the connection process of circuit elements and solar cells [11,12]. However, with the rapid consumption of electronic products and photovoltaic modules, the environmental pollution caused by the related wastes, especially lead, is becoming increasingly serious. In response to concerns about the environment and human health, a number of countries have launched a series of policies and legislations to ban the use of lead. For example, the European Union introduced two draft proposals named the Waste Electrical and Electronic Equipment (WEEE) and the Reduction of Hazardous Substances (RoHS) directives [13,14,15]. Japan required all new electronic/electrical products to be lead free. [16] The United States and the Republic of Korea have also enacted relevant laws [17,18,19]. So far, an increasing number of countries have made great efforts to look for lead-free alternatives to tin–lead alloy solders. Lead-free solders [20,21,22], such as Sn–Cu, Sn–Zn, and so forth, have received considerable attention [23,24]. They are not only environmentally friendly but have also proved to be attractive alternatives to some extent [25]. However, lead-free solders cannot meet all standards, including wettability, low melting point, mechanical integrity, and so forth [26,27]. Electrically conductive adhesives (ECAs) are regarded as the most promising alternative to tin–lead alloy solders because they are more environmentally friendly than lead, easier to fabricate, and have a lower processing temperature. Moreover, they can provide higher-resolution printing compared with tin–lead alloy solders [28].

ECAs mainly consist of an organic/polymer matrix and electrically conductive fillers, which provide the mechanical and electrical properties for ECAs, respectively. Generally, both thermosetting and thermoplastic materials can be used as the polymer matrix of ECAs, such as epoxy resin [29], silicone [30], phenolic epoxy [31], and so forth. Possible electrically conductive fillers include silver [32,33,34,35,36,37,38,39,40,41,42,43], gold [44,45,46], nickel [47,48,49], copper [50,51,52], graphene, and nanocarbon black [53,54,55,56,57]. Among the various electrically conductive fillers, Ag flakes are widely used in current commercial ECAs because they show high electrical conductivity and are easily processible. Note that over 50 wt % conductive fillers should be used generally in ECAs to achieve high electrical conductivity. Therefore, an increasing number of researchers have devoted themselves to studying electrically conductive fillers, in particular, reducing the mass fraction of Ag used in ECAs while ensuring electrical conductivity. Ji et al. [58] developed an ECA with a low Ag content based on the ternary hybrid of Ag microflakes, Ag nanospheres, and nanowires. Xu et al. fabricated a silver nanowire/graphene nanocomposite ECA via a self-assembly method [59]. In summary, much progress has been achieved regarding electrically conductive fillers in ECAs.

The conventional preparation process of ECAs consists of the following steps: preparing the polymer matrix and electrically conductive fillers, respectively, and mixing these two materials by stirring and rolling. Throughout the entire process, secondary pollution and mass loss are inevitably caused. At the same time, the stirring operation introduces many small and stable bubbles, which affect the electrical conductivity of the ECAs [60]. Therefore, the shortcomings of the conventional preparation process of ECAs should be remedied.

Powder spraying technology, which is mostly applied in the mechanical engineering field, provides an approach to fix the powder to substrates [61]. It can eliminate the vigorous mixing process of different components during processing of the target material, thus causing little disturbance to polymer matrix and avoiding secondary pollution and mass loss. Hence, in this study, we introduced powder spraying technology into the preparation of ECAs, whereby silver flakes are sprayed into epoxy resin to produce ECAs. This process results in excellent electrical conductivity and mechanical properties compared with the conventional method.

## 2. Experiment

### 2.1. Raw Materials and Experimental Equipment

#### 2.1.1. Raw Materials

The following raw materials were used as supplied: Ag flakes (FAg-8921) from Sino-Platinum Metals Co., Ltd. (Kunming, China); bisphenol-F-based epoxy resin (EPON 862) with an epoxy equivalent weight of 165–173 g/eq, a viscosity of 2.5–4.5 Paˑs (25 °C), and a density of 1.17 × 10^6^ g/m^3^ from Shell (China) Co., Ltd. (Beijing, China); a curing agent (amine adduct, PN-40) with an average particle size of about 10–12 μm, a softening point of 105–110 °C, and a pot life of about 3 months from Japanese Ajinomoto Co., Ltd. (Kawasaki, Japan); 2-ethoxyethyl acetate (AR, 98%) from Shanghai Aladdin Biochemical Technology Co., Ltd. (Shanghai, China); a powder spray bottle from Hopeck Plastics Industry (Suzhou) Co., Ltd. (Suzhou, China); and round cover slips (10 mm in diameter) from Jianghai Glass Instrument Factory (Jiangsu, China).

#### 2.1.2. Experiment Equipment

Electrical resistivity measurement was performed with an RTS-9 four-point probe resistivity measuring instrument (Four Probes Tech Co., Ltd., Guangzhou, China). Lap shear strength measurement was performed with a CMT 6503 electromechanical universal testing machine (Sans Testing Machine Co., Ltd., Shenzhen, China). SEM was performed with a JEOL (JSM-7900F, JEOL, Kyoto, Japan) high-resolution scanning electron microscope.

### 2.2. Preparation of ECAs

#### 2.2.1. Powder Spraying Process

The fabrication procedure consisted of three main steps: preparation of the polymer matrix, spraying of Ag flakes, and heat curing. The bisphenol-F-based epoxy resin (EPON 862) was used for the polymer matrix, which showed low viscosity and good chemical resistance characteristics. PN-40 was used as the latent curing agent, which reacts with epoxy resins at high temperatures on heating. First, 1 g of epoxy resin with 0.2 g of curing agent (PN-40) was stirred by hand clockwise with a glass rod (1.2–2.0 laps/s) for 7 min; then, 0.5 g of 2-ethoxyethyl acetate was added and stirred for 3 min at room temperature and atmospheric pressure. Second, the sample was vacuumed for 30 min at room temperature. Note that the vacuum process conducted at room temperature cannot lead to the gelation reaction of the epoxy resin system since PN-40 is a latent curing agent. Thus, the polymer matrix was obtained.

For the spraying of Ag flakes process, the polymer matrix was first coated on the substrate. Second, the Ag flakes were sprayed into the polymer matrix with the mass fraction of the Ag flakes ranging from 0 to 80 wt % and then cured for 1 h at 150 °C in an oven. The whole preparation time was within 2 h. After that, it cooled down to room temperature for 10 h and the ECAs fabricated by the powder spraying process were obtained.

#### 2.2.2. Conventional Process

For comparison with the powder spraying process, the conventional process was also used in this study. The fabrication procedure was as follows: First, the polymer matrix was prepared with the same method as the powder spraying process. Second, the Ag flakes were added into the polymer matrix with their mass fraction ranging from 0 to 80 wt %, with stirring performed as mentioned in the powder spraying process for 5 min simultaneously. After the stirring operation, it was coated on the substrate and cured for 1 h at 150 °C in an oven. The whole preparation time was within 2 h. After it cooled down to room temperature for 10 h, the ECAs fabricated by the conventional process were obtained.

### 2.3. Experiment Tests

#### 2.3.1. Tests on Electrical Conductivity

To test the electrical conductivity, round cover slips of 10 mm in diameter were used as the substrate for the ECAs. The prepared ECAs were coated on the round cover slips. After curing and cooling down to room temperature, the electrical resistivity of the ECAs was measured with an RTS-9 four-point probe resistivity measurement meter. Each point was chosen five times, and the average value was taken for further data processing.

#### 2.3.2. Tests on Shear Strength

The tests on shear strength were carried out using a CMT 6503 electromechanical universal testing machine employing the rigid-to-rigid tension loading mode. LY12-CZ aluminum alloy plates (Mingtai aluminium industry Co., Ltd., Zhengzhou, China) (100 × 25 × 2 mm^3^) were used as substrates for the ECAs for shear strength testing. They were first polished with abrasive paper and rinsed with acetone, then dried at room temperature. Afterward, the ECAs with a weight of 0.03–0.09 g were bonded between two LY12-CZ aluminum alloy plates with a contact area of 25 × 25 mm^2^ and a thickness of about 0.2–0.5 mm. In the tests, polyimide tape (0.3 mm in width, 0.07 mm in thickness) was used to control the thickness of the ECAs. It was adhered to the aluminum alloy plates and the thickness was controlled by the layers of the polyimide tape. Specifically, for the powder spraying process, the bond steps of the ECAs on LY12-CZ aluminum alloy plates were as follows: the polyimide tape was adhered to the aluminum alloy plates; the as-prepared polymer matrix was coated with a thin blade; the Ag flakes, ranging from 0 to 80 wt %, were sprayed; another aluminum alloy plate was covered and the two aluminum alloy plates were fixed with two binder clips on both sides. These operations were done at room temperature and atmospheric pressure, followed by curing at 150 °C for 1 h and cooling down to room temperature for 10 h. For the conventional process, the bond steps were similar to the powder spraying process to some extent: the polyimide tape was adhered to the aluminum alloy plates; the as-prepared mixture of polymer matrix was coated with Ag flakes ranging from 0 to 80 wt % with a thin blade; another aluminum alloy plate was covered and the two aluminum alloy plates were fixed with two binder clips on both sides. These operations were done at room temperature and atmospheric pressure, followed by curing at 150 °C for 1 h and cooling down to room temperature for 10 h. After cooling down to room temperature, the polyimide tape adhered to the aluminum alloy plates was removed in both the powder spraying process and conventional process. An electromechanical universal testing machine was used to test the lap shear strength of the ECAs coated between two aluminum alloy plates with a speed of 10 mm/min. The maximum load was measured when shear failure occurred. Each point was chosen five times, and the average value was taken for further data processing. The shear strength can be calculated with the following formula:

(1) $$\tau =\frac{P}{B\cdot L}$$

where τ is the shear strength of the ECAs (MPa), P is the maximum load when the sample fails (N), B is the width of the sample lap joint surface (mm), and L is the length of sample lap surface (mm).

## 3. Results and Discussion

### 3.1. Electrical Property of ECAs

In order to investigate the electrical property of the ECAs prepared with the powder spraying process and the conventional process, a series of experiments were carried out with an RTS-9 four-point probe resistivity measuring instrument.

**Figure 1:**
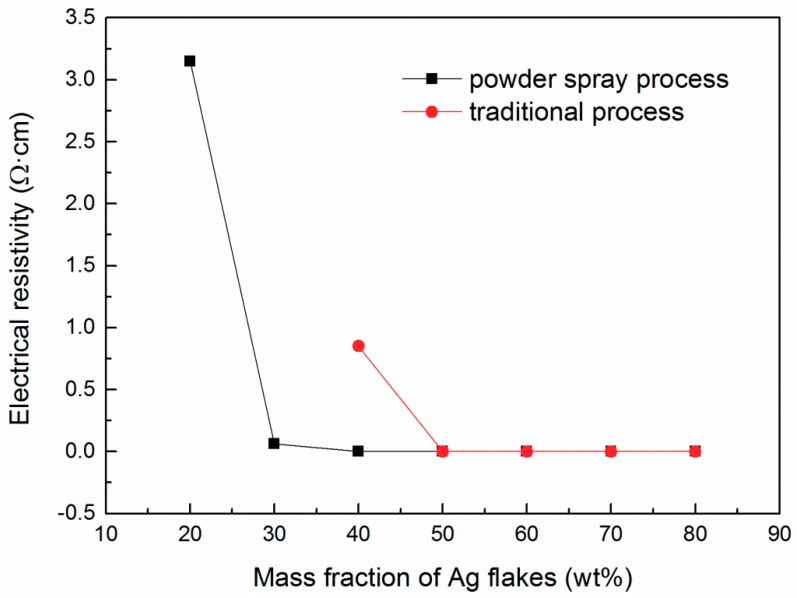
Electrical property of electrically conductive adhesives (ECAs) fabricated by the powder spraying process and the conventional process.

Figure 1 shows the electrical property of the ECAs fabricated by the powder spraying process and the conventional process. As shown in the figure, the electrical resistivity for both ECAs exhibited a similar tendency of decreasing with a mass fraction of Ag flakes below 50 wt % and remaining stable with a mass fraction of Ag flakes higher than 50 wt %. This indicates that the electrical conductivity has a positive relationship with the content of Ag flakes. It can be clearly seen that the electrical resistivity of ECAs fabricated by the powder spraying process was lower than that of ECAs by the conventional process. Especially, ECAs with the conventional process showed no value of electrical conductivity under the mass fraction of Ag flakes below 40 wt %, while the ECAs with the powder spraying process showed obvious electrical conductivity. As the mass fraction of Ag flakes grew to 40 wt %, the ECAs fabricated by the powder spraying process exhibited a remarkable electrical resistivity of 1.9 × 10^−3^ Ωˑcm. However, the electrical resistivity of ECAs with the conventional process was 0.85 Ωˑcm, which was almost 400 times that of ECAs fabricated with the powder spraying process. This proves that the powder spraying process is feasible and better than the conventional process.

The results can be explained as follows. As mentioned above, epoxy resin was used as the polymer matrix. The epoxy resin, as the main component of the ECAs, fully dispersed Ag flakes when the mass fraction of the Ag flakes was below 40 wt % when the conventional process was adopted. The vast majority of Ag flakes were wrapped with epoxy resin after even stirring in the conventional process, leading to Ag flakes not being able to complete the circuit; consequently, there was no electrical resistivity value for the ECAs. However, during the powder spraying process, Ag flakes were sprayed into the epoxy resin and freely contacted with each other, so the ECAs prepared with the powder spraying process showed remarkable electrical conductivity. Therefore, ECAs fabricated with the powder spraying process showed excellent electrical conductivity compared with those fabricated by the conventional process.

### 3.2. Mechanical Property

In order to investigate the mechanical property of ECAs prepared by the powder spraying process and the conventional process, a series of experiments were carried out with an electromechanical universal testing machine.

**Figure 2:**
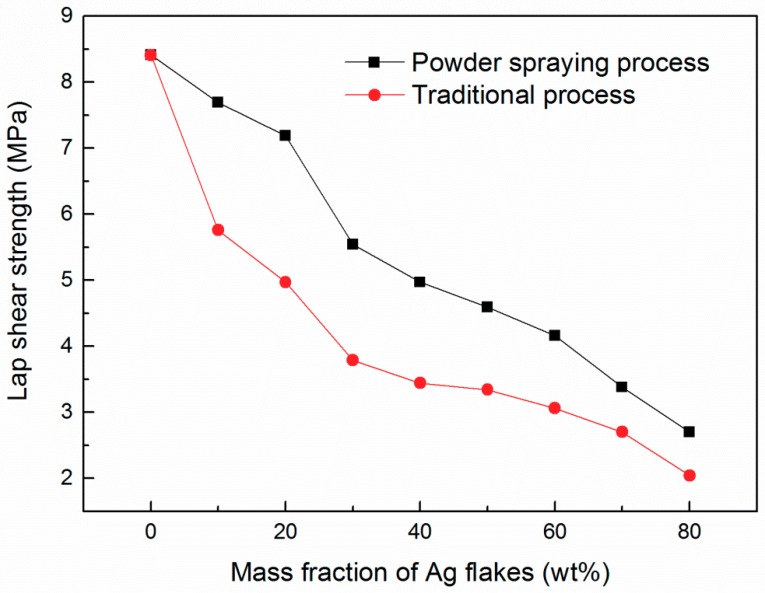
Lap shear strength of the ECAs fabricated by the powder spraying process and the conventional process.

Figure 2 demonstrates the lap shear strength of ECAs fabricated by the two different methods. Both ECAs fabricated by the two different methods showed a similar trend. As the mass fraction of Ag flakes increased, the lap shear strength of the ECAs simultaneously decreased. As the mass fraction of Ag flakes increased, the bonding strength between the ECAs and the substrate decreased. This indicates that the epoxy resin provided the mechanical property. Meanwhile, the figure shows that the ECAs fabricated by the powder spraying process had higher lap shear strength than those by the conventional process.

### 3.3. SEM Analysis

In order to investigate the morphology of ECAs prepared by the powder spraying process and the conventional process, high-resolution SEM was used to observe the microstructure of Ag flakes and ECAs, with the content of Ag flakes ranging from 20 to 80 wt %.

#### 3.3.1. Ag Flakes

**Figure 3:**
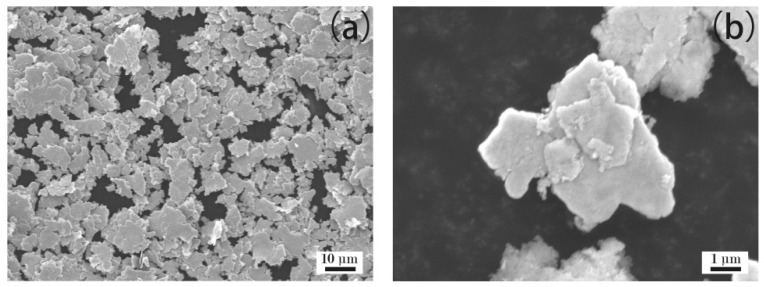
SEM images of Ag flakes. (**a**): aggregates of Ag flakes; (**b**): magnification image of Ag flakes.

Figure 3 shows the microstructure of the Ag flakes. It can be observed that the Ag flakes had an irregular flake structure, with their size ranging from several to a dozen microns.

#### 3.3.2. ECAs Filled with 20 wt % Ag Flakes

**Figure 4:**
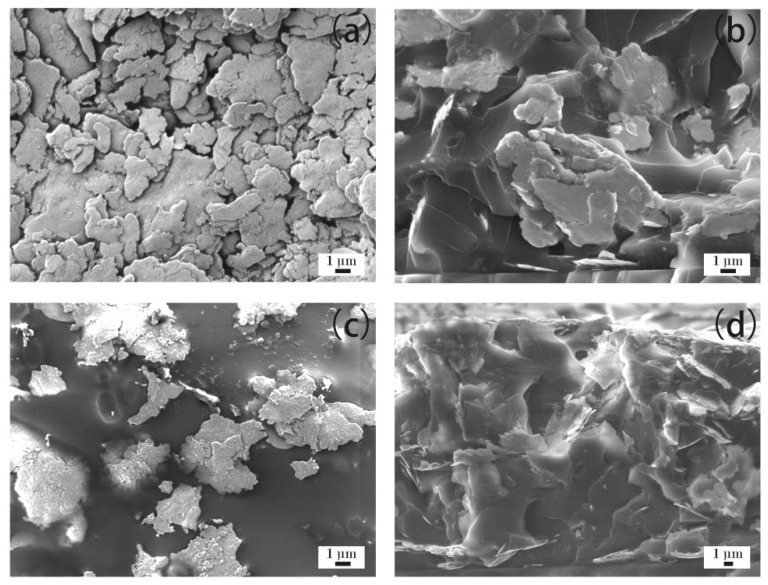
ECAs filled with 20 wt % Ag flakes. (**a**) ECAs fabricated by the powder spraying process; and (**b**) cross sections of ECAs fabricated by the powder spraying process; (**c**) ECAs fabricated by the conventional process; and (**d**) cross sections of ECAs fabricated by the conventional process.

Figure 4a–d show the microstructure of the ECAs filled with 20 wt % Ag flakes. Figure 4a,b show ECAs fabricated by the powder spraying process, and Figure 4c,d show the ECAs fabricated by the conventional process. From Figure 4a, it can be observed that the Ag flakes gathered together via interconnecting sheets and formed a network in the powder spraying process. Therefore, it could complete a circuit and showed electrical conductivity, as shown in Figure 1. Figure 4b shows its cross-sectional morphology. A few Ag flakes with an irregular flake structure are shown scattered in the epoxy resin because of the low mass fraction of Ag flakes.

Compared with the powder spraying process shown in Figure 4a,b, the Ag flakes were more discretely scattered in the epoxy resin shown in Figure 4c,d for the conventional process. The discrete Ag flakes of the ECAs could not form a network. This illustrates the reason for the ECAs fabricated by the conventional process not being able to show electrical conductivity, as shown in Figure 1, with the Ag flakes with a mass fraction of 20 wt %.

#### 3.3.3. ECAs Filled with 30 wt % Ag Flakes

**Figure 5:**
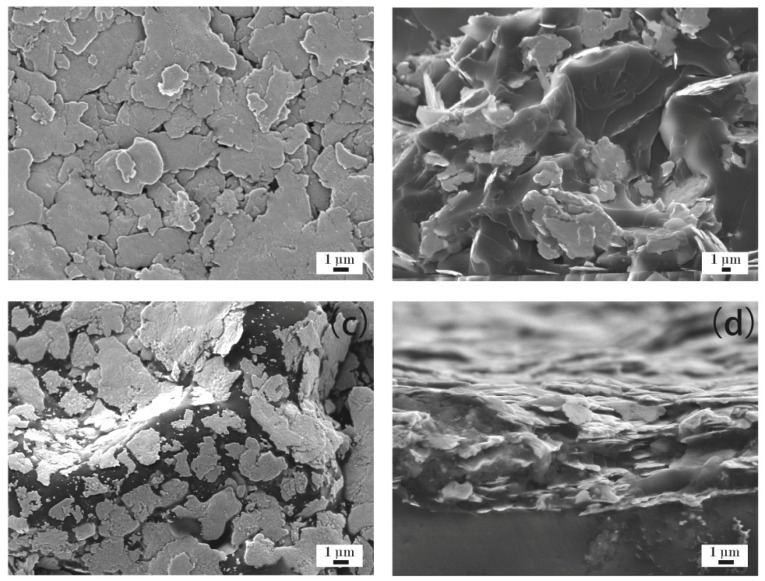
ECAs filled with 30 wt % Ag flakes. (**a**) ECAs fabricated by the powder spraying process; and (**b**) cross sections of ECAs fabricated by the powder spraying process; (**c**) ECAs fabricated by the conventional process; and (**d**) cross sections of ECAs fabricated by the conventional process.

Figure 5a–d show the microstructure of the ECAs filled with 30 wt % Ag flakes. Figure 5a,b show the ECAs fabricated by the powder spraying process, and Figure 5c,d show the ECAs fabricated by the conventional process. From Figure 5a, it can be observed that a dense network of Ag flakes was formed compared with that shown in Figure 4a. Also, Figure 5b shows more Ag flakes fixed into the polymer matrix than that of Figure 4b. Therefore, the ECAs filled with 30 wt % Ag flakes had higher electrical conductivity than those filled with 20 wt % Ag flakes.

Compared with the ECAs filled with 20 wt % Ag flakes (Figure 4c,d), there were more Ag flakes scattered in the epoxy resin, as shown in Figure 5c,d, for the conventional process. However, the increasing content of Ag flakes could not yet form a network. This illustrates the reason for the ECAs fabricated by the conventional process not being able to show electrical conductivity, as shown in Figure 1, with the Ag flakes with a mass fraction of 30 wt %.

#### 3.3.4. ECAs Filled with 40 wt % Ag Flakes

**Figure 6:**
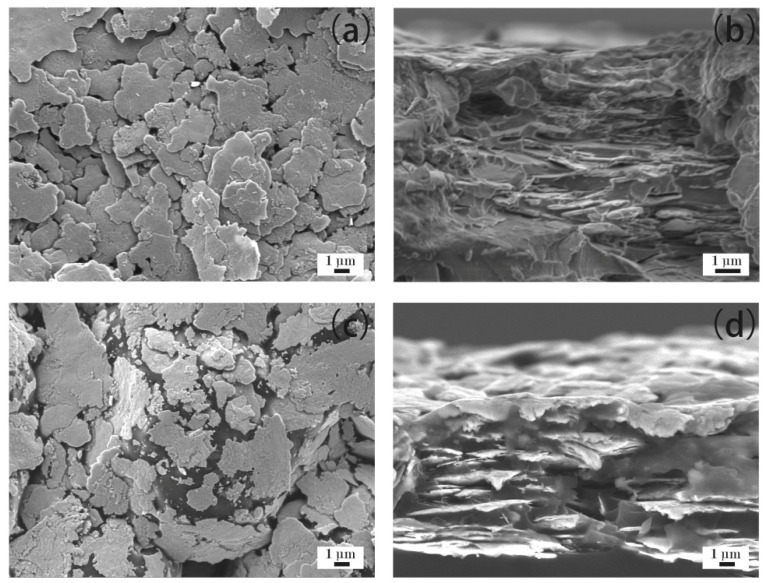
ECAs filled with 40 wt % Ag flakes. (**a**) ECAs fabricated by the powder spraying process; and (**b**) cross sections of ECAs fabricated by the powder spraying process; (**c**) ECAs fabricated by the conventional process; and (**d**) cross sections of ECAs fabricated by the conventional process.

Figure 6a–d show the microstructure of ECAs filled with 40 wt % Ag flakes. Figure 6a,b show the ECAs fabricated by the powder spraying process, and Figure 6c,d show the ECAs fabricated by the conventional process. As shown in Figure 6a, a dense network of Ag flakes was formed compared with that shown in Figure 5a. Also, Figure 6b shows more Ag flakes fixed into the polymer matrix than that of Figure 5b. Therefore, the ECAs filled with 40 wt % Ag flakes had higher electrical conductivity than those filled with 30 wt % Ag flakes.

An incomplete network of Ag flakes emerged, as shown in Figure 6c. However, the Ag flakes shown in Figure 6c were still discrete and could insufficiently contact with each other, though more Ag flakes were fixed into the polymer matrix compared with that in Figure 5c. Meanwhile, the Ag flakes in the cross section are denser in Figure 6d than those in Figure 5d. Therefore, the ECAs filled with 40 wt % Ag flakes by the conventional process showed electrical conductivity to some extent with high electrical resistivity, as shown in Figure 1.

#### 3.3.5. ECAs Filled with 50 wt % Ag Flakes

**Figure 7:**
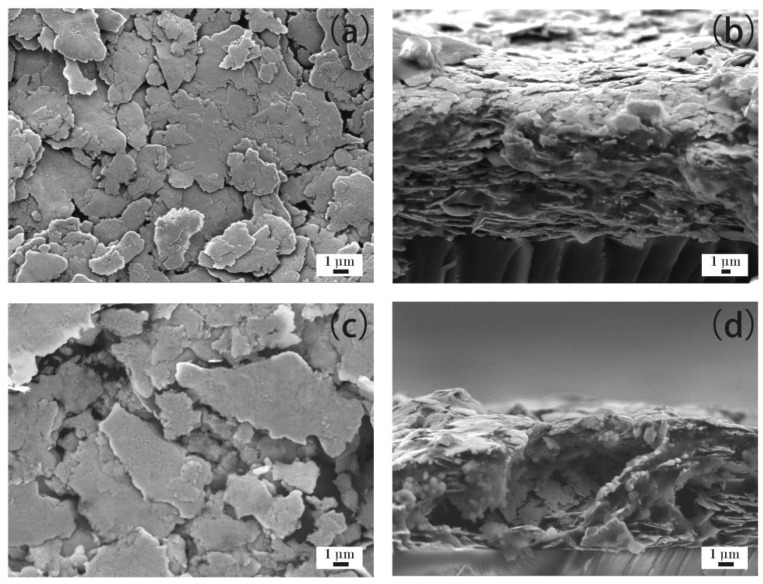
ECAs filled with 50 wt % Ag flakes. (**a**) ECAs fabricated by the powder spraying process; and (**b**) cross sections of ECAs fabricated by the powder spraying process; (**c**) ECAs fabricated by the conventional process; and (**d**) cross sections of ECAs fabricated by the conventional process.

Figure 7a–d show the microstructure of the ECAs filled with 50 wt % Ag flakes. Figure 7a,b show the ECAs fabricated by the powder spraying process, and Figure 7c,d show the ECAs fabricated by the conventional process. As shown in Figure 7a,b, there were more Ag flakes fixed into the polymer matrix compared with that shown in Figure 6a,b. Meanwhile, a denser network was formed (Figure 7a) compared with that shown in Figure 6a.

A network of Ag flakes was basically formed (Figure 7c). The Ag flakes shown in Figure 7c relatively closely contacted with each other compared with those in Figure 6c. Meanwhile, the Ag flakes in the cross section were denser (Figure 7d) compared with those shown in Figure 6d. Therefore, the ECAs filled with 50 wt % Ag flakes by the conventional process showed obvious electrical conductivity compared with those filled with 40 wt % Ag flakes, as shown in Figure 1. Furthermore, the ECAs filled with 50 wt % Ag flakes had higher electrical conductivity than those filled with 40 wt % Ag flakes via both methods, which agrees well with the electrical resistivity data shown in Figure 1.

#### 3.3.6. ECAs Filled with 60 wt % Ag Flakes

**Figure 8:**
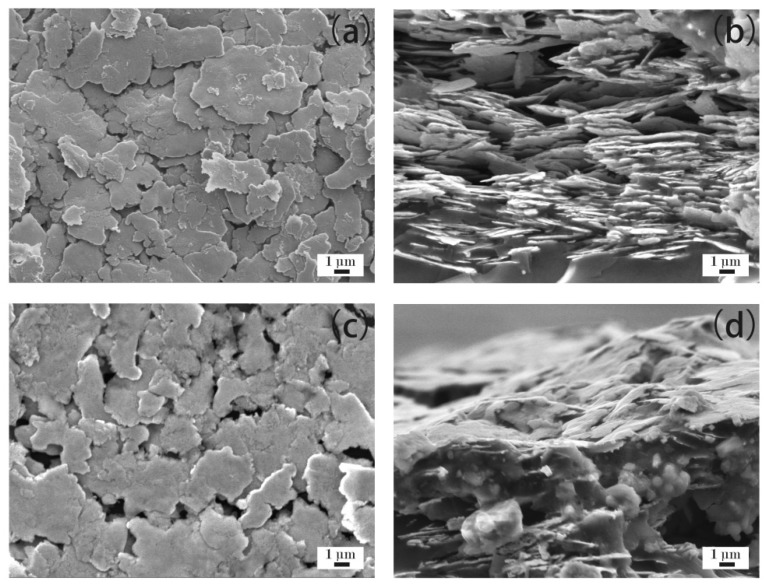
ECAs filled with 60 wt % Ag flakes. (**a**) ECAs fabricated by the powder spraying process; and (**b**) cross sections of ECAs fabricated by the powder spraying process; (**c**) ECAs fabricated by the conventional process; and (**d**) cross sections of ECAs fabricated by the conventional process.

Figure 8a–d show the microstructure of ECAs filled with 60 wt % Ag flakes. Figure 8a,b show the ECAs fabricated by the powder spraying process, and Figure 8c,d show the ECAs fabricated by the conventional process. As shown in Figure 8a,b, there were more Ag flakes fixed into the polymer matrix compared with Figure 7a,b. Meanwhile, as Figure 8a shows, the network formed with Ag flakes was denser than that in Figure 7a. Simultaneously, there were more Ag flakes fixed into the polymer matrix, as shown in Figure 8c,d, compared with that in Figure 7c,d. Also, the network of Ag flakes was fully formed, as shown in Figure 8c, by the conventional process. Thus, when the content of Ag flakes was 60 wt %, the ECAs fabricated by the two methods showed good electrical resistivity, as shown in Figure 1.

#### 3.3.7. ECAs Filled with 70 wt % Ag Flakes

**Figure 9:**
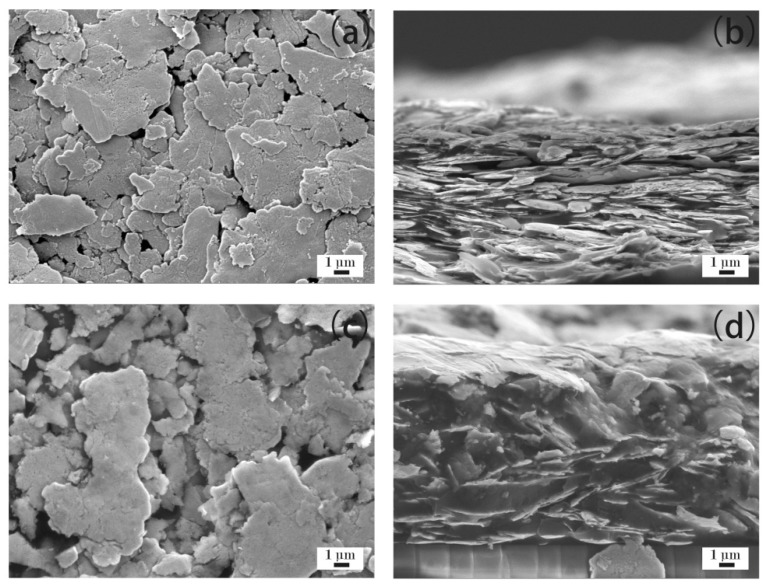
ECAs filled with 70 wt % Ag flakes. (**a**) ECAs fabricated by the powder spraying process; and (**b**) cross sections of ECAs fabricated by the powder spraying process; (**c**) ECAs fabricated by the conventional process; and (**d**) cross sections of ECAs fabricated by the conventional process.

Figure 9a–d show the microstructure of the ECAs filled with 70 wt % Ag flakes. It can be seen that the network formed with the Ag flakes became increasingly dense as the content of Ag flakes increased. There was a rare gap where the connection of Ag flakes was disconnected. This demonstrates that the Ag flakes adequately contacted with each other when the content of Ag flakes reached up to 70 wt % in both ECAs made by the two different methods. This is consistent with the electrical resistivity results (Figure 1).

#### 3.3.8. ECAs Filled with 80 wt % Ag Flakes

**Figure 10:**
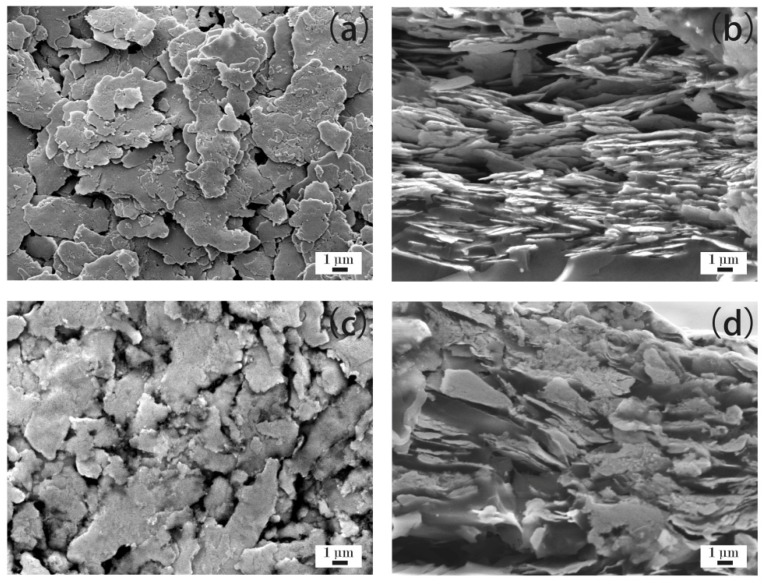
ECAs filled with 80 wt % Ag flakes. (**a**) ECAs fabricated by the powder spraying process; and (**b**) cross sections of ECAs fabricated by the powder spraying process; (**c**) ECAs fabricated by the conventional process; and (**d**) cross sections of ECAs fabricated by the conventional process.

Figure 10a–d show the microstructure of the ECAs filled with 80 wt % Ag flakes. It can be seen that the network formed with Ag flakes reached the densest state as the content of Ag flakes was up to 80 wt %. There was a rare gap where the connection of Ag flakes was disconnected. This demonstrates that the Ag flakes as electrical conduct fillers achieved compact interconnection when the content of Ag flakes reached up to 80 wt % in both ECAs prepared by the two different methods. This is consistent with the electrical resistivity results (Figure 1).

## 4. Conclusions

In this study, we developed a novel preparation method of ECAs by the powder spraying process. The ECAs fabricated by the powder spraying process exhibited excellent properties compared with those made by the conventional preparation process. Especially at a low content of Ag flakes, they showed good electrical conductivity, while the ECAs fabricated by the conventional process could not. At the 40 wt % mass fraction of Ag flakes, ECAs fabricated by the powder spraying process exhibited a remarkable electrical resistivity of 1.9 × 10^−3^ Ωˑcm, which was almost 400 times that of the ECAs fabricated by the powder spraying process. Further, the lap shear strength of the ECAs fabricated by the powder spraying process was higher than that of the conventional process. Therefore, the powder spraying process is feasible and superior to the conventional process. It can be speculated that the powder spraying process may have potential applications in the fields of solar panels, electrode materials, supercapacitors, and so on.
